# DegS protease regulates the motility, chemotaxis, and colonization of *Vibrio cholerae*

**DOI:** 10.3389/fmicb.2023.1159986

**Published:** 2023-04-05

**Authors:** Mei Zou, Kaiying Wang, Jiajun Zhao, Huifang Lu, Hui Yang, Meirong Huang, Lu Wang, Guangli Wang, Jian Huang, Xun Min

**Affiliations:** ^1^Department of Laboratory Medicine, Affiliated Hospital of Zunyi Medical University, Zunyi, Guizhou, China; ^2^School of Laboratory Medicine, Zunyi Medical University, Zunyi, Guizhou, China; ^3^Department of Blood Transfusion, Affiliated Hospital of Zunyi Medical University, Zunyi, Guizhou, China

**Keywords:** *Vibrio cholerae*, DegS, motility, chemotaxis, colonization

## Abstract

In bacteria, DegS protease functions as an activating factor of the σ^E^ envelope stress response system, which ultimately activates the transcription of stress response genes in the cytoplasm. On the basis of high-throughput RNA sequencing, we have previously found that *degS* knockout inhibits the expression of flagellum synthesis- and chemotaxis-related genes, thereby indicating that DegS may be involved in the regulation of *V. cholerae* motility. In this study, we examined the relationships between DegS and motility in *V. cholerae*. Swimming motility and chemotaxis assays revealed that *degS* or *rpoE* deletion promotes a substantial reduction in the motility and chemotaxis of *V. cholerae*, whereas these activities were restored in *ΔdegS::degS* and *ΔdegSΔrseA* strains, indicating that DegS is partially dependent on σ^E^ to positively regulate *V. cholerae* activity. Gene-act network analysis revealed that the cAMP–CRP–RpoS signaling pathway, which plays an important role in flagellar synthesis, is significantly inhibited in *ΔdegS* mutants, whereas in response to the overexpression of *cyaA/crp* and *rpoS* in the *ΔdegS* strain, the motility and chemotaxis of the *ΔdegS* + *cyaA*/*crp* and *ΔdegS* + *rpoS* strains were partially restored compared with the *ΔdegS* strain. We further demonstrated that transcription levels of the flagellar regulatory gene *flhF* are regulated by DegS *via* the cAMP–CRP–RpoS signaling pathway. Overexpression of the *flhF* gene in the *ΔdegS* strain partially restored motility and chemotaxis. In addition, suckling mouse intestinal colonization experiments indicated that the *ΔdegS* and *ΔrpoE* strains were characterized by the poor colonization of mouse intestines, whereas colonization efficacy was restored in the *ΔdegSΔrseA*, *ΔdegS + cyaA/crp*, *ΔdegS + rpoS*, and *ΔdegS + flhF* strains. Collectively, our findings indicate that DegS regulates the motility and chemotaxis of *V. cholerae via* the cAMP–CRP–RpoS–FlhF pathway, thereby influencing the colonization of suckling mouse intestines.

## Introduction

*Vibrio cholerae* is classified into more than 200 serogroups based on surface O-antigens, among which the O1 and O139 serotypes are characterized as pathogenic strains responsible for cholera epidemics and pandemics, whereas non-O1/non-O139 *V. cholerae* (NOVC) produce no cholera toxins and consequently do not cause cholera (Lekshmi et al., 2018). However, NOVC are now recognized as pathogens responsible for sporadic and local infectious outbreaks (Xie et al., 2020), in which they can cause gastrointestinal infection (Lee et al., 2007), as well as extra-intestinal disease, such as bacteremia (Li et al., 2020b; Wang et al., 2022), meningitis (Hao et al., 2015), bacterial emphysema (Marinello et al., 2017), and have even been associated with significant mortality (Engel et al., 2016). Although in recent years, an increasing number of cases of NOVC infection have been reported, the pathogenic mechanisms of non-O1/non-O139 *V. cholerae* have yet to be sufficiently established. Bacterial motility, intestinal colonization, and cholera toxin secretion have been established to be the three most important factors associated with the pathophysiology of *V. cholerae*, and it is accordingly reasoned that reducing these virulence factors could make an important contribution to the prevention and treatment of cholera (Charles and Ryan, 2011). Bacterial motility has currently been identified as a virulence determinant in *V. cholerae*, which has a unipolar flagellum and is dependent on chemotaxis and motility to initiate appropriate virulence gene expression during the early stages of infection (Martinez et al., 2009; Matilla and Krell, 2018; Subramanian and Kearns, 2019). Within the host, *V. cholerae* typically utilizes its flagellum to cross the protective layer of the intestinal mucosa and enter the microvilli of intestinal epithelial cells, wherein it attaches to and colonizes the epithelial cells (Almagro-Moreno et al., 2015). When exposed to environmental selection pressure, *V. cholerae* can also escape from the site of colonization and enter the external environment by controlling its motility (Nielsen et al., 2006).Non-motile mutants of *V. cholerae* have been observed to be less virulent than the wild-type (WT) strain in young mouse model (Syed et al., 2009), whereas in humans, non-motile mutants of live attenuated *V. cholerae* vaccines are characterized by reduced reactivity (Coster et al., 1995). Consequently, motility plays essential roles in both the life cycle and pathogenicity of *V. cholerae*, and thus from the perspective of cholera prevention and treatment, it would be beneficial to study the molecular basis of *V. cholerae* motility and establish means whereby this motility could be impaired.

DegS is a serine protease that plays an important role in regulation of the σ^E^ (*rpoE*) stress response (de Regt et al., 2015). Interestingly, on the basis of RNA sequencing analysis, we previously found that *degS* knockout inhibited the expression of flagellum synthesis- and chemotaxis-related genes, and the findings of gene-act network analysis provided evidence to indicate that the cAMP–CRP–RpoS signaling pathway is significantly inhibited in the *degS* mutant (Huang et al., 2019a). Cyclic adenylate (cAMP), a second messenger involved in the regulation of cell function, binds to its signal transduction receptor (CRP) and plays important roles not only in a range of catabolic functions but also in flagellar synthesis, toxin production, and other non-catabolic processes (Manneh-Roussel et al., 2018). There are two predicted CRP binding sites in the vicinity of the *rpoS* promoter, and the cAMP–CRP complex can bidirectionally regulate *rpoS* expression at different stages of development (Cheng and Sun, 2009). The findings of previous research have revealed that deletion of *rpoS* markedly reduces the expression of several flagellar synthesis and chemotactic genes, which accordingly impairs bacterial motility and chemotaxis (Hengge, 2009). In the present study, we found that trends in the expression of nine flagellum synthesis- and chemotaxis-related genes are affected by *degS* knockout in a similar manner to seven genes affected by *rpoS* knockout. Furthermore, we established that the motility of a *ΔdegS* mutant on motility plates is less pronounced than that of the WT strain, thereby indicating that DegS may regulate the motility of *V. cholerae*. In this study, we systematically investigate the effects of DegS on the motility of *V. cholerae*.

Regulation of the expression of genes associated with flagellar synthesis is currently a particularly active area of focus in dynamics research, and also a critical link in dynamic regulation (Echazarreta and Klose, 2019; Khan et al., 2020). In this regard, the flagellar genes of *V. cholerae* have been established to be transcribed in a four-tiered transcriptional hierarchy (Prouty et al., 2001). Among these genes, *flhF*, which is transcribed in a class II operon and positively regulates class III gene transcription (Kazmierczak and Hendrixson, 2013; Arroyo-Pérez and Ringgaard, 2021), encodes a membrane-associated signal recognition particle family GTP-binding protein (FlhF), which restricts flagellum assembly to the cell pole (Green et al., 2009). In *V. cholerae*, a deficiency in *flhF* can result in flagellum loss (Correa et al., 2005). In this study, we established that DegS modulates the expression of the flagellum regulatory gene *flhF via* the cAMP–CRP–RpoS signaling pathway, thereby influencing the motility, chemotaxis, and colonization of *V. cholerae*.

## Materials and methods

### Bacterial strains and growth conditions

As a wild-type strain in this study, we used non-O1/non-O139 *V. cholerae* HN375, which was obtained from the China Center for Type Culture Collection (accession number CCTCC AB209168; Luo et al., 2011). *Escherichia coli* DH5α and DH5α-λpir were used for cloning and WM3064 was used as the donor strain in sexual pilus conjugation assay. All strains were grown in Luria–Bertani (LB) medium at 37°C until reaching the exponential stage of growth stage. The medium was supplemented with the following antibiotic or arabinose concentrations as needed: 100 μg/ml ampicillin, 50 μg/ml chloramphenicol, and 0.1% arabinose. Supplementary Table 1 lists all strains used in this study.

### DNA manipulation and genetic techniques

All deletion mutants were constructed from the HN375 wild-type strain using the suicide plasmid pWM91 (Wu et al., 2015). The primers used for amplification are listed in Supplementary Table 2. To facilitate complementation, the coding region of *crp* or *cyaA* was cloned into plasmid pBAD24 and then sexually pilus conjugated into *Δcrp* or *ΔcyaA*, to generate the complemented strains *Δcrp::crp* and *ΔcyaA::cyaA*, respectively. Similar methods were used to construct the *ΔdegSΔrseA::rseA*, *ΔrpoE::rpoE*, and *ΔrpoS::rpoS* strains. To produce overexpressing strains, the recombinant plasmid pBAD24-*crp* or pBAD24-*cyaA* was used to transform *ΔdegS* strains *via* electroporation to yield the *ΔdegS + crp-* or *ΔdegS + cyaA-*overexpressing strain, respectively. Similar methods were used to construct the *ΔdegS + rpoS*, *ΔdegS + mcp*, and *ΔdegS + flhF* strains. To generate double overexpression strains, the *crp* coding sequence was cloned into a pBAD33 plasmid and used the transform the *ΔdegS + cyaA* overexpressing strain *via* electroporation. To induce gene expression, all complementary and overexpressing strains were cultured in LB medium supplemented with 0.1% arabinose.

### Quantitative real-time PCR assay

Having reached the exponential stage of growth (OD_600_ = 0.6), bacterial cultures were harvested by centrifugation at 8,000 *rpm* for 5 min. Total RNA was extracted from the pelleted cells using TRIzol reagent and reverse transcribed to generate cDNA using PrimeScript™ RT reagent Kit with gDNA Eraser (Takara, China). The concentration and purity of RNA and cDNA were determined. qRT-PCR was performed using TB Green Premix Ex Taq™II (Huang et al., 2019a). For each experimental group, analyses were performed in triplicate.

### Swimming motility assay

After bacterial cultures had reached the exponential stage of growth (OD_600_ = 0.6), cell suspensions were used to inoculate motility plates containing 0.25% agar, 1% tryptone, and 0.5% NaCl. After culturing for 14 h at 37°C, the diameters of the swimming zones around the sites of inoculation were measured at 2-h intervals. All experiments were performed in triplicate.

### Chemotaxis assay

The capillary chemotactic assay performed in this study was based on a modified version of that previously described (Gordillo et al., 2007; Elgamoudi and Korolik, 2022). After bacteria had reached the exponential stage of growth, cell cultures were centrifuged at 1,200 × *g* for 2 min. The resulting cell pellets were washed twice with chemotaxis buffer (PH = 7.0) consisting of 10.0 ml 1 M KPO_4_, 0.2 ml 0.5 M EDTA, 3.915 g NaCl, 0.1 ml 10 mM methionine, 1.0 ml 10 M lactic acid, 1.0 L ddH_2_O, and adjusted to OD_600_ = 0.3. A 300-μl aliquot of the bacterial suspension was drawn into the needle cap of a 1-ml syringe, and 200 μl of a chemotaxis solution [chemotaxis buffer was used as a blank control (Partridge et al., 2019)] was aspirated into the syringe. After laying on a horizontal surface for 1 h, the movement of bacteria into the syringe was measured by quantifying the number of bacteria that accumulated by random motility or chemotaxis. The relative chemotaxis index (RCI) was calculated as the ratio between the numbers of bacteria entering the test syringe and those in the control syringe (Gordillo et al., 2007). All experiments were performed in triplicate.

### Suckling mouse colonization assay

An intestinal colonization model was generated using 6-day-old CD1 suckling mice. All animal experiments performed in this study were approved by the Ethics Committee of Zunyi Medical University. Suckling mice were assigned randomly to each experimental group (*n* = 8 animals per group) and maintained in a specific pathogen-free environment. After the bacteria had reached the exponential stage of growth, the cells were collected by centrifugation at 1,200 × *g* for 2 min. The pellets thus obtained were washed twice with 1× phosphate-buffered saline, and the bacterial suspension was adjusted to OD_600_ = 0.5 and then diluted 100 times to a concentration of 1 × 10^7^ CFU per ml. Each suckling mouse received 50 μl (~5 × 10^5^ CFU) of cell suspensions *via* gavage, with the same volume of 1× phosphate-buffered saline serving as a baseline control (Sarkar et al., 2018; Wang et al., 2018; Cho et al., 2022). The inoculation dose was determined through several pre-experimental explorations, and we found that higher concentration including 5 × 10^7^ CFU per 50 μl and 5 × 10^6^ CFU per 50 μl of bacterial suspension resulted in massive death of the suckling mice in 18 h, while 5 × 10^5^ CFU per 50 μl did not. After 18 h of observation, the intestines of sacrificed mice were dissected out, weighed, and ground. Having diluted gut preparation 100-fold, 50-μL aliquots were spread on LB plates supplemented with 0.01 mg/ml streptomycin, and colonies were counted after incubation at 37°C for 18 h. The results were expressed as the logarithm of CFUs/g intestine (cfu/g).

### Statistical analysis

Data are presented as the means ± standard deviation. The data were analyzed using a two-way analyses of variance (ANOVA) and two-tailed unpaired *t*-tests. Two-way ANOVA was performed in conjunction with Šídák’s multiple comparison test, where the independent variable was time (h) and the dependent variable was diameter (mm).

## Results

### The positive regulation of *Vibrio cholerae* motility by DegS is partially dependent on σ^E^

**Figure 1:**
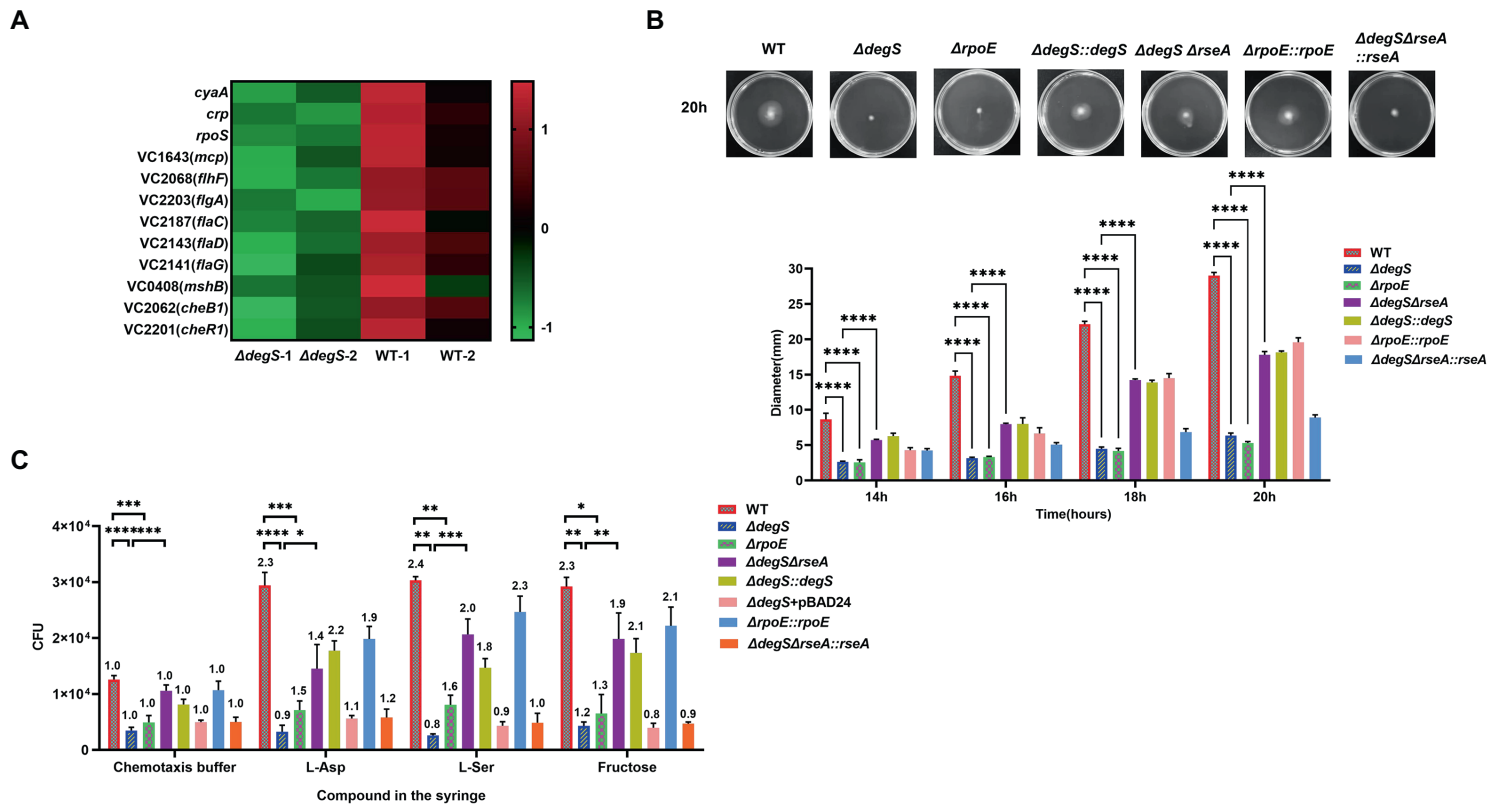
The positive regulation of *Vibrio cholerae* motility by DegS is partially dependent on σ^E^. **(A)** RNA-Seq analysis of differentially expressed genes in *ΔdegS* and wild-type (WT) strains reveled that *crp*, *cyaA*, *rpoS,* and nine motility-related genes were downregulated in *ΔdegS* strains. **(B)** The swimming motility of the WT, *ΔdegS*, *ΔdegS::degS*, *ΔrpoE*, *ΔrpoE::rpoE*, *ΔdegSΔrseA*, and *ΔdegSΔrseA::rseA* strains were assessed on soft agar plates. The representative pictures for the swimming zones are obtained at 20 h. Data were analyzed using a two-way ANOVA. ****, *p* < 0.0001 between two strains. **(C)** Analysis of the chemotactic responses to aspartate, serine, and fructose by the WT, *ΔdegS*, *ΔdegS::degS*, *ΔdegS* + pBAD24, *ΔrpoE*, *ΔrpoE::rpoE*, *ΔdegSΔrseA*, and *ΔdegSΔrseA::rseA* strains. Error bars indicate the SDs based on three replicated experimental values (*n* = 3). *, *p* < 0.05; **, *p* < 0.01; ***, *p* < 0.001; and ****, *p* < 0.0001 between two strains (two-tailed unpaired *t*-tests). Numbers on top of each bar indicate the relative chemotactic indexes (RCI).

Our transcriptome sequencing data revealed that *degS* knockdown resulted in a significant down-regulation of nine motility-related gene (Figure 1A; Supplementary Table 3), implying that DegS may influence *V. cholerae* motility. To verify this conjecture, we carried out swimming motility assays, and found that the diameter of the swimming zone of the *degS* mutant on motility plates was significantly smaller than that of the WT strain (Figure 1B; Supplementary Figure 1). Subsequently, we performed a chemotaxis assay using three widely utilized attractants, namely 50 mM fructose (Liu et al., 2020b), 100 mM serine (l-ser; Roggo et al., 2018), and 100 μM aspartic acid (l-Asp; Long et al., 2017). Either with or without attractant, the motility of the *ΔdegS* strain was found to be lower than that of the WT strain. However, motility was partially restored in the *ΔdegS::degS* strain, although not in the empty vector strain (*ΔdegS +* pBAD24; Figure 1C). These observations thus provided evidence that DegS positively regulates the motility of *V. cholerae*.

It has previously been established that the essential function of DegS is to provide active σ^E^ for cells by degrading RseA (Alba et al., 2001), and the absence of DegS has been demonstrated to result in a significant reduction in σ^E^ activity (Ades et al., 1999). In contrast, the absence of RseA coincides with a high constitutive activity σ^E^ (De Las Peñas et al., 1997; Missiakas et al., 1997). To investigate whether RpoE is involved in the DegS-regulated motility of *V. cholerae*, we constructed a *rpoE* mutant (*ΔrpoE*), a *degS* and *rseA* double-knockout strain (*ΔdegSΔrseA*) and the corresponding complemented strains (*ΔrpoE::rpoE* and *ΔdegSΔrseA::rseA*). qRT-PCR results confirmed that expression of the *rpoE* gene in these strains was consistent with the aforementioned results (Supplementary Figure 2). The *ΔrpoE* strain showed a significant decrease in swimming motility and chemotaxis compared to the WT strain, and the *ΔrpoE::rpoE* strain could revert to near WT strain levels. Whereas the *ΔdegSΔrseA* strain showed swimming motility and chemotaxis close to WT strain levels, the *ΔdegSΔrseA::rseA* strain showed phenotypic characteristics similar to the *ΔdegS* strain (Figures 1B,C; Supplementary Figure 1). These findings accordingly indicate that the positive regulation of *V. cholerae* motility *via* DegS is partially dependent on σ^E^.

### Regulation of *Vibrio cholerae* motility by DegS may involve the co-regulation of cAMP and CRP

**Figure 2:**
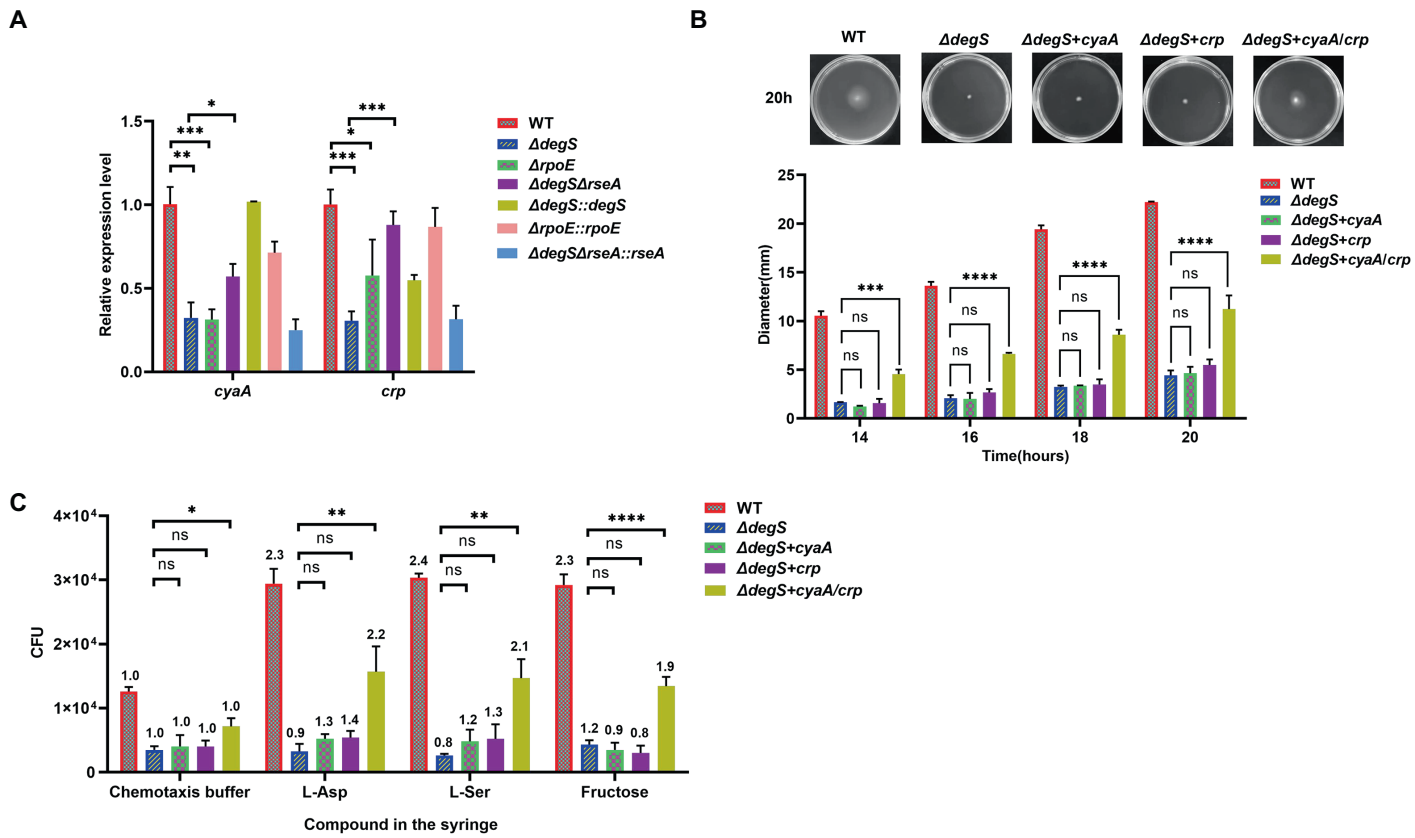
The regulation of *Vibrio cholerae* motility by DegS may involve the co-regulation of cAMP and CRP. **(A)** The mRNA levels of *cyaA* and *crp* in the WT, *ΔdegS*, *ΔdegS::degS*, *ΔrpoE*, *ΔrpoE::rpoE*, *ΔdegSΔrseA*, and *ΔdegSΔrseA::rseA* strains. Statistical analyses were performed using an unpaired *t*-test. *, *p* < 0.05; **, *p* < 0.01; and ***, *p* < 0.001 between two strains. **(B)** The swimming motility of WT, *ΔdegS*, *ΔdegS* + *crp*, *ΔdegS* + *cyaA* and *ΔdegS* + *cyaA*/*crp* strains was determined. The representative pictures for the swimming zones are obtained at 20 h. Statistical analyses were performed using a two-way ANOVA. ***, *p* < 0.001 and ****, *p* < 0.0001 between two strains; ns, not significant. **(C)** Analysis of the chemotactic responses to aspartate, serine, and fructose for the WT, *ΔdegS*, *ΔdegS* + *crp*, *ΔdegS* + *cyaA*, and *ΔdegS* + *cyaA*/*crp* strains. Error bars indicate the SDs based on three replicated experimental values (n = 3). *, *p* < 0.05; **, *p* < 0.01; and ****, *p* < 0.0001 between two strains; ns, not significant (unpaired *t*-tests). Numbers on top of each bar indicate the relative chemotactic indexes (RCI).

Previous studies have shown that the cAMP–CRP complex regulates flagellum biosynthesis (Liu et al., 2020a). Our transcriptome data revealed that the levels of *cyaA* and *crp* transcripts were significantly lower in the *ΔdegS* strain (Figure 1A). We further verified these observations by qRT-PCR and found that DegS and RpoE positively regulate the expression of *cyaA* and *crp* (Figure 2A). *CyaA* encodes adenylyl cyclase, which can synthesize cAMP from ATP, and *crp* encodes the cyclic adenylyl receptor protein (CRP; Zhang et al., 2013). To gain a better understanding of the role of cAMP and CRP in the DegS-mediated regulation of *V. cholerae* motility, we overexpressed *cyaA* or *crp* in *ΔdegS* mutants (*ΔdegS* + *cyaA* and *ΔdegS* + *crp*) and performed swimming and chemotaxis assays, the results of which revealed that overexpression of neither *cyaA* nor *crp* could compensate for the lost motility and chemotaxis of *ΔdegS* strains (Figures 2B,C; Supplementary Figure 3). In most cases, cAMP and CRP are mutually dependent on each other for functional activity (Kolb et al., 1993; Busby and Ebright, 1999), and when both *cyaA* and *crp* were overexpressed in the *ΔdegS* strains (*ΔdegS* + *cyaA*/*crp*), we found that the motility and chemotaxis of the *ΔdegS* + *cyaA*/*crp* strain could be partially restored (Figures 2B,C). These findings indicate that the regulation of *V. cholerae* motility by DegS may involve the co-regulation of cAMP and CRP.

### RpoS participates in the DegS-mediated regulation of *Vibrio cholerae* motility

**Figure 3:**
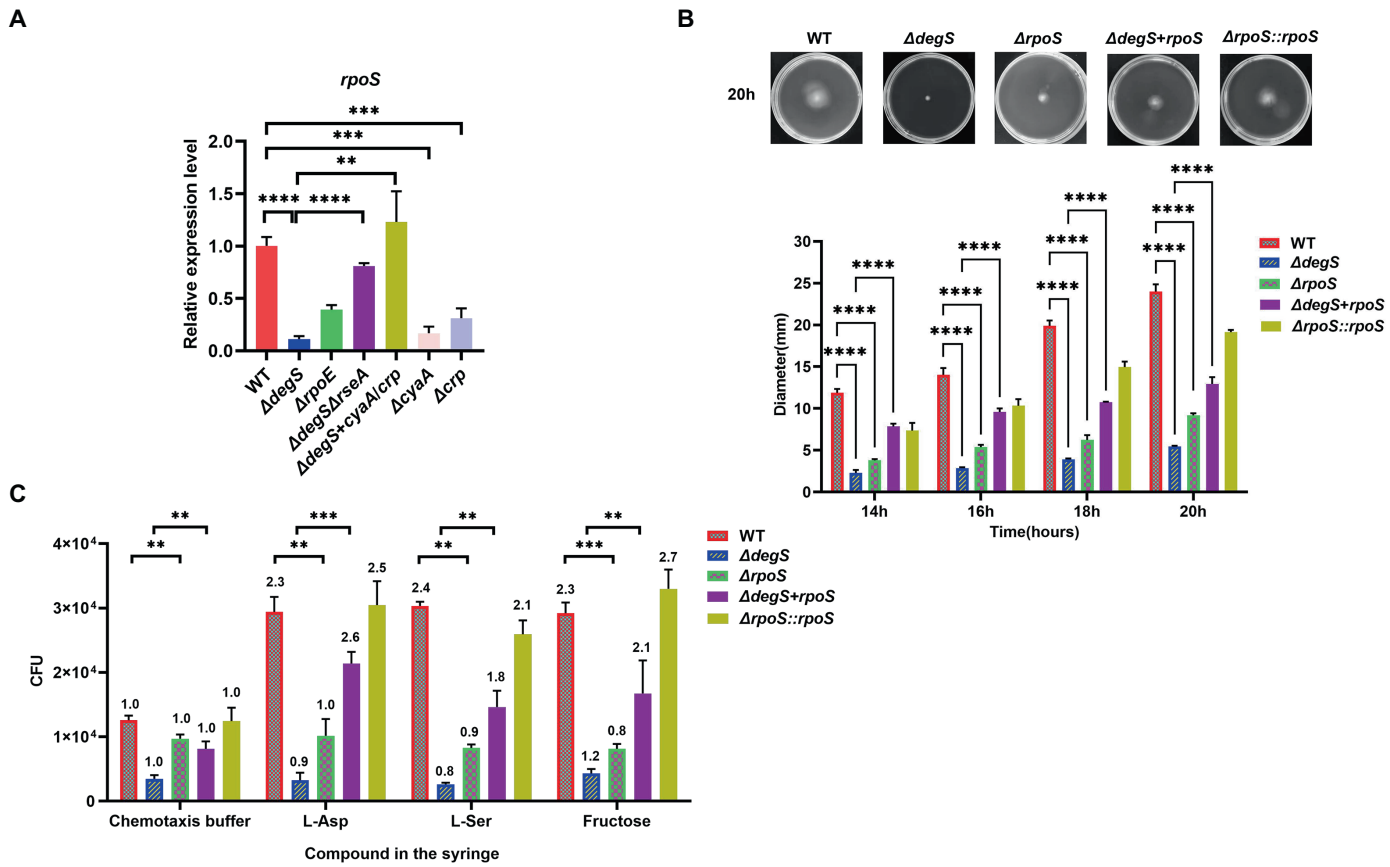
RpoS participates in the DegS-mediated regulation of *Vibrio cholerae* motility. **(A)** The mRNA levels of *rpoS* in WT, *ΔdegS*, *ΔrpoE*, *ΔdegSΔrseA*, *ΔcyaA*, *Δcrp*, and *ΔdegS* + *cyaA*/*crp* strains (unpaired *t*-tests). **(B)** The swimming motility of WT, *ΔdegS*, *ΔrpoS*, *ΔrpoS::rpoS*, and *ΔdegS* + *rpoS* strains was assessed. The representative pictures for the swimming zones are obtained at 20 h. Statistical analyses were performed using a two-way ANOVA. ***, *p* < 0.001 and ****, *p* < 0.0001 between two strains. **(C)** Analysis of the chemotactic responses to aspartate, serine, and fructose for WT, *ΔdegS*, *ΔrpoS*, *ΔrpoS::rpoS*, and *ΔdegS* + *rpoS* strains. Error bars indicate the SDs based on three replicated experimental values (*n* = 3). **, *p* < 0.01; ***, *p* < 0.001 (unpaired *t*-tests). Numbers on top of each bar indicate the relative chemotactic indexes (RCI).

Knockout of the *rpoS* gene has been demonstrated to markedly reduce the expression of several flagellum synthesis and chemotactic genes, which impairs bacterial motility and chemotaxis (Hengge, 2009). The cAMP–CRP complex has been characterized as a transcriptional activator of *rpoS* (Guo et al., 2015), and the results of our qRT-PCR analyses indicated that *degS* positively affects the transcriptional levels of *rpoS via cyaA* and *crp* (Figure 3A). To examine the role of RpoS in the DegS-mediated regulation of motility in *V. cholerae*, we generated *rpoS* knockout, *rpoS* compensation and *rpoS* overexpression strains (*ΔrpoS*, *ΔrpoS::rpoS* and *ΔdegS* + *rpoS*). Compared with WT strain, the motility and chemotaxis of *ΔrpoS* mutant were significantly reduced, whereas these activities were partially compensated in the *ΔrpoS::rpoS* and *ΔdegS* + *rpoS* strains (Figures 3B,C; Supplementary Figure 4). These results thus tend to indicate that RpoS participates in the DegS-mediated regulation of *V. cholerae* motility.

### The effect of DegS on *Vibrio cholerae* motility may be involved in the expression of FlhF

**Figure 4:**
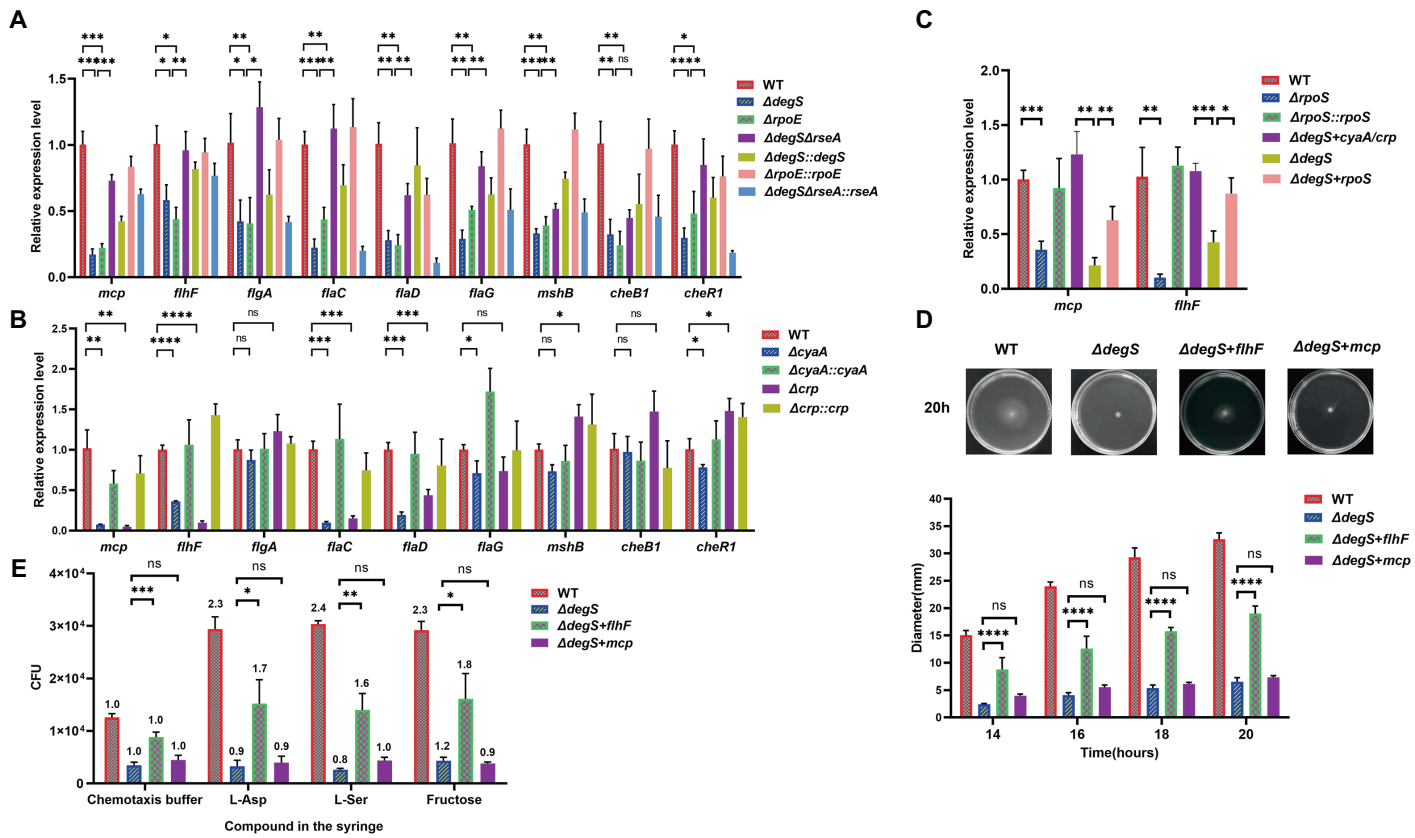
The effect of DegS on *Vibrio cholerae* motility may involve the expression of FlhF. **(A,B)** The mRNA levels of nine flagellum synthesis- and chemotaxis-related genes in WT, *ΔdegS*, *ΔrpoE*, *ΔrpoE::rpoE*, *ΔdegS ΔrseA*, *ΔdegS ΔrseA::rseA*, *ΔdegS::degS*, *ΔcyaA*, *ΔcyaA::cyaA*, *Δcrp*, and *Δcrp::crp* strains (unpaired *t*-tests). **(C)** The mRNA levels of *mcp* and *flhF* in WT, *ΔdegS*, *ΔrpoS*, *ΔrpoS::rpoS*, *ΔdegS* + *cyaA*/*crp*, and *ΔdegS* + *rpoS* strains (unpaired *t*-tests). **(D)** The swimming motility of WT, *ΔdegS*, *ΔdegS* + *flhF*, and *ΔdegS* + *mcp* strains was assessed. The representative pictures for the swimming zones are obtained at 20 h. Statistical analyses were performed using a two-way ANOVA. ****, *p* < 0.0001 between two strains; ns, not significant. **(E)** The analysis of chemotactic responses to aspartate, serine, and fructose for WT, *ΔdegS*, *ΔdegS* + *flhF*, and *ΔdegS* + *mcp* strains. Error bars indicate the SDs based on three replicated experimental values (*n* = 3). *, *p* < 0.05; **, *p* < 0.01; ***, *p* < 0.001; ns, not significant (unpaired *t*-tests). Numbers on top of each bar indicate the relative chemotactic indexes (RCI).

Our transcriptome data revealed that compared with the WT strain, nine flagellar synthesis and chemotaxis genes were significantly repressed in *ΔdegS* strains, namely, VC1643 (*mcp*), VC2068 (*flhF*), VC2203 (*flgA*), VC2187 (*flaC*), VC2143 (*flaD*), VC2141 (*flaG*), VC0408 (*mshB*), VC2062 (*cheB1*), and VC2201 (*cheR1*; Figure 1A). Furthermore, our qRT-PCR results confirmed that the deletion of either *degS* or *rpoE* significantly inhibited the expression of these nine genes, and the compensation of *degS* or *rpoE* could restore the transcription levels of these genes (Figure 4A). To further establish which of these genes might participate in the DegS regulation of *V. cholerae* motility, we determined the transcriptional levels of the nine dynamic-related genes in *ΔcyaA*, *Δcrp*, and other strains. Compared with the WT strain, the transcription levels of the *mcp*, *flhF*, *flaC*, and *flaD* genes were substantially reduced in both the *ΔcyaA* and *Δcrp* strains, whereas levels were found to be restored in the *ΔcyaA::cyaA* and *Δcrp::crp* strains (Figure 4B). In addition, the transcription of these four genes was partially restored in the *ΔdegAΔrseA* strain, whereas the transcription of these four genes was suppressed by the compensation of *rseA* (Figure 4A). In *V. cholerae*, FlaC, and FlaD have been identified as components of the flagellum filament, although appear to be non-essential for filament synthesis and motility (Rui et al., 2010). Contrastingly, MCP and FlhF play important roles in the motility of bacterial flagella (Huang et al., 2019b; Arroyo-Pérez and Ringgaard, 2021). Moreover, RpoS has been shown to be required for the expression of both *mcp* and *flhF* (Nielsen et al., 2006). Our qRT-PCR analysis in the present study revealed that the transcription levels of *mcp* and *flhF* indeed positively regulated by RpoS, and the levels were partially recovered in the *ΔdegS* + *cyaA*/*crp* and *ΔdegS* + *rpoS* strains (Figure 4C), thereby indicating that DegS may control the transcription of *mcp* and *flhF via* the cAMP–CRP–RpoS pathway. To further assess whether FlhF and MCP play roles in the DegS regulation of *V. cholerae* motility, we, respectively, overexpressed proteins in the *ΔdegS* mutant and performed swimming and chemotaxis assays. The results revealed that motility and chemotaxis were partially restored in the *ΔdegS* + *flhF* strain, although not in the *ΔdegS* + *mcp* strain (Figures 4D,E; Supplementary Figure 5). Taken together, these observations indicate that the DegS-mediated regulation of *V. cholerae* motility may be involved in the regulation of FlhF expression.

### DegS affects intestinal colonization by *Vibrio cholerae via* the cAMP–CRP–RpoS–FlhF pathway

**Figure 5:**
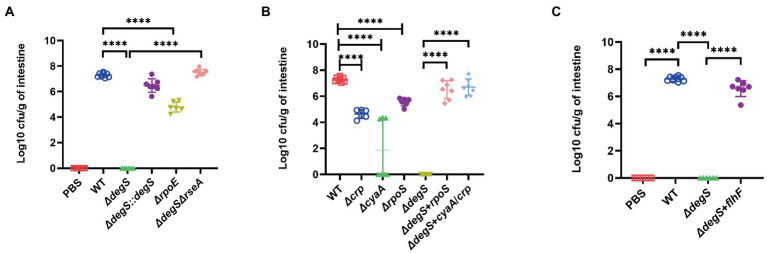
DegS influences *Vibrio cholerae* intestinal colonization *via* the cAMP–CRP–RpoS–FlhF pathway. **(A–C)** The numbers of bacteria in the intestine of suckling mice after infection with 1 × 10^7^ colony-forming units of the WT, *ΔdegS*, *ΔdegS::degS*, *ΔrpoE*, *ΔdegSΔrseA*, *ΔcyaA*, *Δcrp*, *ΔrpoS*, *ΔdegS* + *rpoS*, *Δdegs* + *cyaA*/*crp*, *and ΔdegS* + *flhF* strains for 18 h. The results are expressed as the logarithm of colony-forming units/g intestine (cfu/g; ±SD, *n* = 8). ****, *p* < 0.0001 between two strains (unpaired *t*-tests).

Flagellum-driven motility plays an essential role in bacterial colonization (Baban et al., 2013; Tamar et al., 2016; Kajikawa et al., 2018), and our *in vitro* experiments revealed that DegS affects *V. cholerae* motility *via* the cAMP–CRP–RpoS–FlhF signaling pathway. To establish whether the regulatory mechanism is equally important in intestinal colonization by *V. cholerae*, we performed analyses using an intestinal colonization model in suckling mice. The results revealed the poor colonization ability of the *ΔdegS* and *ΔrpoE* strains, whereas the colonization efficacy of the *ΔdegSΔrseA* strain was comparable to that of the WT strain (Figure 5A), which tends to indicate that the effect of DegS on the intestinal colonization of *V. cholerae* is partially dependent on σ^E^. The colonization ability of *ΔcyaA*, *Δcrp* and *ΔrpoS* was also observed to be lower than that of the WT strain, whereas colonization efficacy was effectively restored in the *ΔdegS* + *cyaA*/*crp*, *ΔdegS* + *rpoS*, and *ΔdegS* + *flhF* strains (Figures 5B,C). These findings indicate that cAMP–CRP, RpoS, and FlhF are involved in the DegS-mediated regulation of *V. cholerae* intestinal colonization.

## Discussion

**Figure 6:**
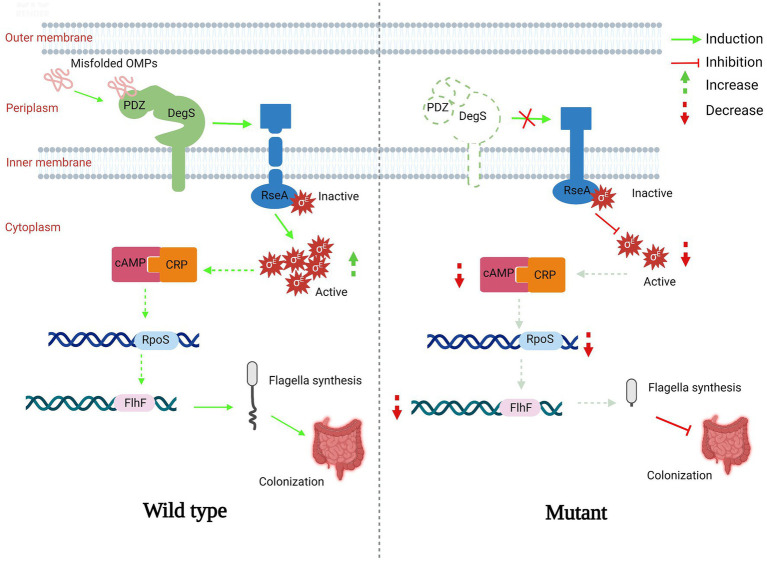
A proposed model showing the mechanisms whereby DegS positively regulates *Vibrio cholerae* motility. In the normal state (wild type), the PDZ domain of DegS binds to a large number of misfolded outer membrane proteins and promotes RseA cleavage to release active σ^E^. Subsequently, the expression of *flhF* is induced *via* the cAMP–CRP–RpoS signaling pathway, resulting in the synthesis of bacterial flagella. In the *degS* knockout state (mutant), RseA cleavage is suppressed, amounts of σ^E^ and cAMP–CRP complex are reduced, and the transcription of *rpoS* and *flhF* is downregulated, resulting in impaired bacterial flagella development and poor colonization ability. A red arrow represents inhibition and a downward-pointing dashed red arrow represents a reduction. A green arrow represents induction and an upward-pointing green dashed arrow represents an increase.

In this study, we showed that DegS protease plays essential roles in *V. cholerae* motility, chemotaxis, and colonization, and proposed a model in which DegS protease regulates expression of the flagellum regulatory gene *flhF via* the cAMP–CRP–RpoS signaling pathway, thereby regulating *V. cholerae* motility and influencing its intestinal colonization capacity (Figure 6). DegS, a membrane-anchored periplasmic protease, plays an important role in the σ^E^-mediated stress response pathway (Sohn et al., 2007; Chaba et al., 2011), and as a pressure sensor protein of the σ^E^-mediated stress response, active DegS promotes RseA cleavage and the release active σ^E^, thereby inducing the expression of σ^E^-dependent genes (Walsh et al., 2003). Multiple transcriptional regulatory systems that are directly controlled by σ^E^ are involved in a wide range of biological processes, including stress responses, virulence, motility, biofilm formation, and quorum sensing (Liang et al., 2021), and previous studies have established that RpoE can promote the motility of *Salmonella enterica* serovar typhi (*S. typhi*; Zhang et al., 2015; Spöring et al., 2018). In the present study, we showed that *V. cholerae* motility is significantly inhibited by the deletion of *degS* or *rpoE*, and that this motility could be partially restored in *ΔdegSΔrseA* strains (Figures 1B,C). On the basis of these observations, we thus speculate that the σ^E^-mediated stress response could be the process linking the deletion of *degS* with impaired motility.

To gain a further understanding of the mechanisms underlying the regulation of *V. cholerae* motility by DegS, we performed RNA-seq analysis to investigate the genes that are differentially expressed between *ΔdegS* and WT strains (Figure 1A), and gene-act network analysis indicated that the cAMP–CRP–RpoS signaling pathway may participate in the DegS-mediated regulation of *V. cholerae* motility (Huang et al., 2019a). cAMP–CRP serves as an important global regulator of the expression of numerous regulators and operons. It has been established that more than 7% of the genes in *E. coli* are regulated by the cAMP–CRP system (Zheng et al., 2004). For example, it has been shown that the cAMP–CRP complex bind directly to a region upstream of FlhDC, the main operon associated flagellar production in *E. coli* (Soutourina et al., 1999; Fahrner and Berg, 2015). In the present study, we found that that cAMP and CRP influence the transcription of certain flagellum biosynthesis-related genes in *V. cholerae* (Figure 4B), and that the impaired motility and chemotaxis of a *degS* mutant could be partly reversed by the overexpression of *cyaA* and *crp* (Figures 2B,C). We accordingly speculate that the cAMP–CRP complex participates in the DegS-mediated regulation of *V. cholerae* motility.

The cAMP–CRP complex and RpoS collaborate to regulate multiple cellular processes (Franchini et al., 2015), and as early as 2002, a close association between cAMP–CRP and RpoS was proposed (Hengge-Aronis, 2002b). Two potential cAMP–CRP binding sites are located upstream and downstream of the *rpoS* promoter (Hengge-Aronis, 2002a), and direct binding of the cAMP–CRP complex to the *rpoS* promoter has been demonstrated in *S. typhi* (Cheng and Sun, 2009). In the present study, qRT-PCR was performed to confirm that the cAMP–CRP system also positively regulates transcription of the *rpoS* gene in *V. cholerae* (Figure 3A). We established that only simultaneous overexpression of *cyaA* and *crp* could restore the levels of *rpoS* transcription in the *degS* knockout strain (Figure 3A), which accordingly leads us to speculate that DegS may regulate the expression of RpoS by regulating the cAMP–CRP complex. RpoS has been identified as an important regulator of flagellum biosynthesis in *Yersinia* (Guan et al., 2015), and in *V. cholerae*, the interaction between RpoS and its antiregulator RssB has been shown to influence motility and colonization ability (Wurm et al., 2017; Wölflingseder et al., 2022). We also established that the motility and colonization of *V. cholerae* are suppressed by the deletion of *rpoS* (Figures 3B,C, 5B). A previous comparison of the genes differentially expressed between the WT and *ΔrpoS* strains of *V. cholerae* has revealed that the transcription of *flhF* is dependent on *rpoS* (Nielsen et al., 2006), and in the present study, we confirmed this association based on qRT-PCR analysis and suggest that RpoS is involved in the transcriptional regulation of *flhF* by DegS (Figure 4C).

It has been established that the flagellar genes in *V. cholerae* are expressed on the basis of a four-tiered transcriptional hierarchy, and that *flhF* is transcribed within a class II operon (Prouty et al., 2001). The findings of previous studies have indicated that the *flhF* mutant of *V. cholerae* lacks a polar flagellum, which results in a significant reduction in bacterial motility (Correa et al., 2005), and we demonstrated that transformation with the pBAD24-*flhF* plasmid could partially compensate for the deficient motility and chemotaxis of the *degS* mutant (Figures 4D,E). FlhF plays roles in the polar targeting of the *V. cholerae* flagellum and promotes flagellum assembly by recruiting the earliest flagellar structural component, the inner-membrane MS-ring protein FliF, to the cell pole (Green et al., 2009). FlhF functions as a positive regulator of class III flagellar promoters involved in the synthesis of the basal bodyhook, motor component (MotY), and “core” flagellin (FlaA; Echazarreta and Klose, 2019). In *Campylobacter jejuni*, FlhF has been shown to bind directly to the *flgI* promoter, which encodes a component of the flagellar P-ring, thereby controlling flagellar biosynthesis (Li et al., 2020a). On the basis of these previous finding, we propose that DegS may influence the motility and chemotaxis of *V. cholerae* by regulating FlhF expression *via* the cAMP–CRP–RpoS signaling pathway. Given that the respective overexpression of *cyaA/crp*, *rpoS*, and *flhF* in *ΔdegS* strains only partially restored the normal phenotypes of the *degS* mutant, we speculate that there may be additional factors involved in the DegS-mediated regulation of motility and chemotaxis in *V. cholerae*. Accordingly, the specific mechanisms whereby DegS regulates the motility of *V. cholerae via fhlF* warrant further analyses.

In order to successfully infect a host, *V. cholerae* is dependent on flagellar motility to facilitate penetration of the host intestinal mucosa, and thereby effectively colonize the host intestines (Butler and Camilli, 2005). Our observations in the present study indicate that the DegS-mediated regulation of *V. cholerae* flagellar motility *via* the cAMP–CRP–RpoS–FlhF pathway may contribute to intestinal colonization (Figure 5). In this regard, it has previously been demonstrated that *Salmonella* might use the σ^E^-dependent cell envelope stress response as a cue to determine the spatiotemporal stage of infection during host colonization (Spöring et al., 2018). Colonization of the intestines of suckling mice by *V. cholerae* has been shown to be reduced by mutation of the *rpoE* gene (Kovacikova and Skorupski, 2002), which is consistent with our findings in this study, in which we showed that the deletion of *degS* or *rpoE* had the effect of suppressing the intestinal colonization capacity of *V. cholerae*, whereas this lost function was partially restored in the *ΔdegSΔrseA* strain (Figure 5A). We therefore speculate that *V. cholerae* may be dependent on a DegS-mediated stress response pathway for effective colonization. Furthermore, the cAMP–CRP signaling pathway has been established to modulates the expression of numerous virulence genes and colonization factors during bacterial host colonization (Kariisa et al., 2015; Manneh-Roussel et al., 2018). In this regard, our findings indicate that the DegS-mediated regulation of *V. cholerae* colonization involves regulation of the flagellum regulatory gene *flhF via* the cAMP–CRP–RpoS signaling pathway.

## Conclusion

In this study, we demonstrate that deletion of the *degS* gene attenuates the motility and chemotaxis of *V. cholerae*, which in turn influences the ability of *V. cholerae* to colonize the small intestine of suckling mice. Moreover, we established that the underlying regulatory process is dependent on *rpoE*. Our findings also indicate that DegS plays important regulatory roles in the motility and chemotaxis *V. cholerae* and that the underlying mechanisms may involve the regulation of FlhF expression *via* the cAMP–CRP–RpoS signaling pathway. Collectively, the findings of this study enhance our current understanding of the biological function of DegS and provide new insights into the regulation of *V. cholerae* motility. However, further details are required regarding the regulation of cAMP–CRP by DegS-σ^E^ and the mechanisms whereby FlhF plays a role in the DegS-mediated regulation of *V. cholerae* motility.

## Data availability statement

The datasets presented in this study can be found in online repositories. The names of the repository/repositories and accession number(s) can be found in the article/Supplementary material.

## Author contributions

JH and XM designed the study. MZ, KW, JZ, HL, HY, and LW performed the experiments. MZ, KW, MH, and GW analyzed the data. MZ and JH wrote the paper. XM and JH conceived and supervised the project. All authors contributed to the article and approved the submitted version.

## Funding

This work was supported by grants from the National Natural Science Foundation of China (No. 32060035) and the Science and Technology Project of Guizhou [Qiankehejichu-ZK[2021]470].

## Conflict of interest

The authors declare that the research was conducted in the absence of any commercial or financial relationships that could be construed as a potential conflict of interest.

## Publisher’s note

All claims expressed in this article are solely those of the authors and do not necessarily represent those of their affiliated organizations, or those of the publisher, the editors and the reviewers. Any product that may be evaluated in this article, or claim that may be made by its manufacturer, is not guaranteed or endorsed by the publisher.
