# Supplementary figures and images for: DegS protease regulates the motility, chemotaxis, and colonization of Vibrio cholerae
Source: Front Microbiol. 2023 Apr 5;14:1159986. doi: 10.3389/fmicb.2023.1159986 (PMC10113495; doi:10.3389/fmicb.2023.1159986)

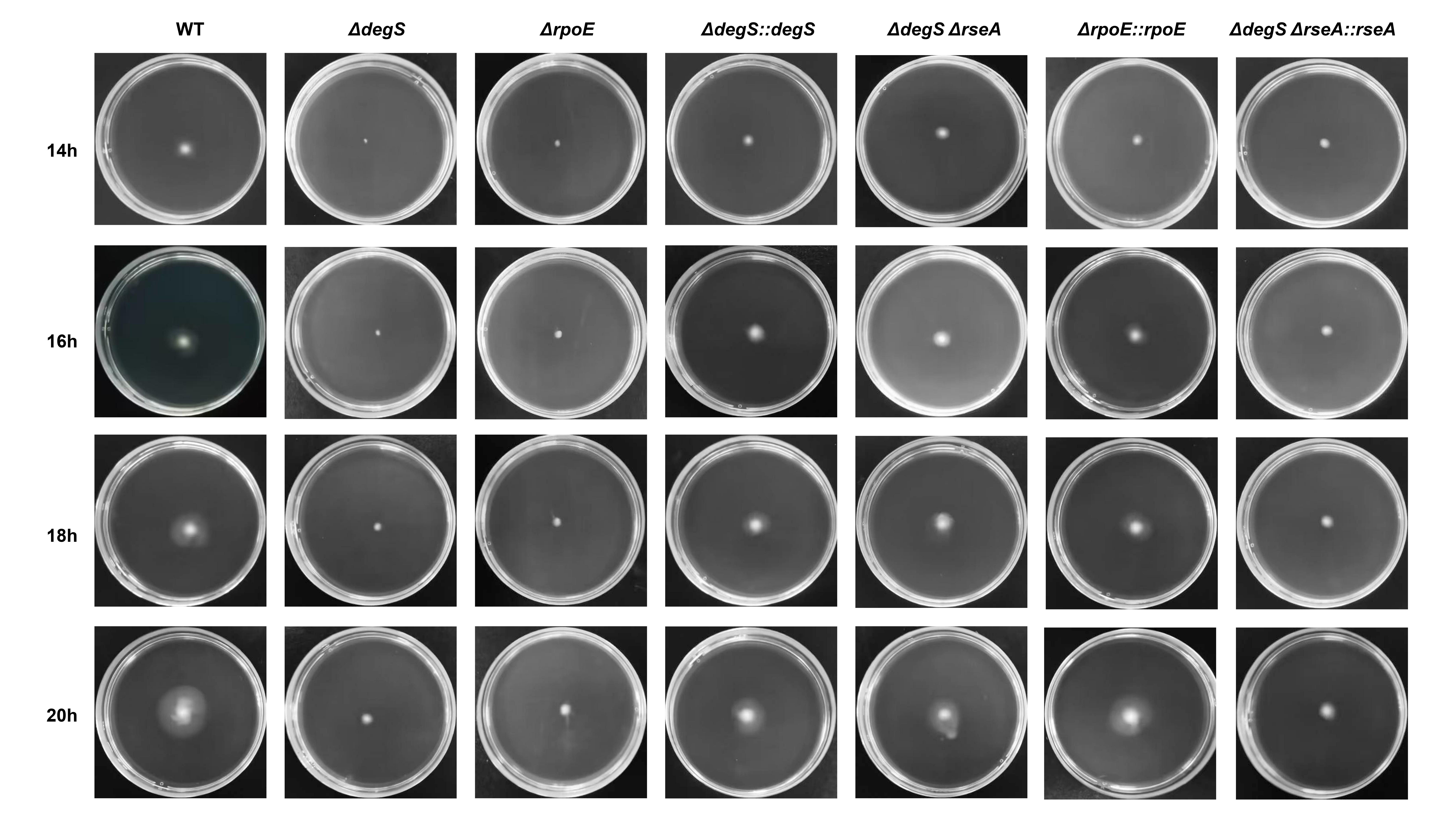

Supplement: Supplementary file 1 [file Image_1.TIF]

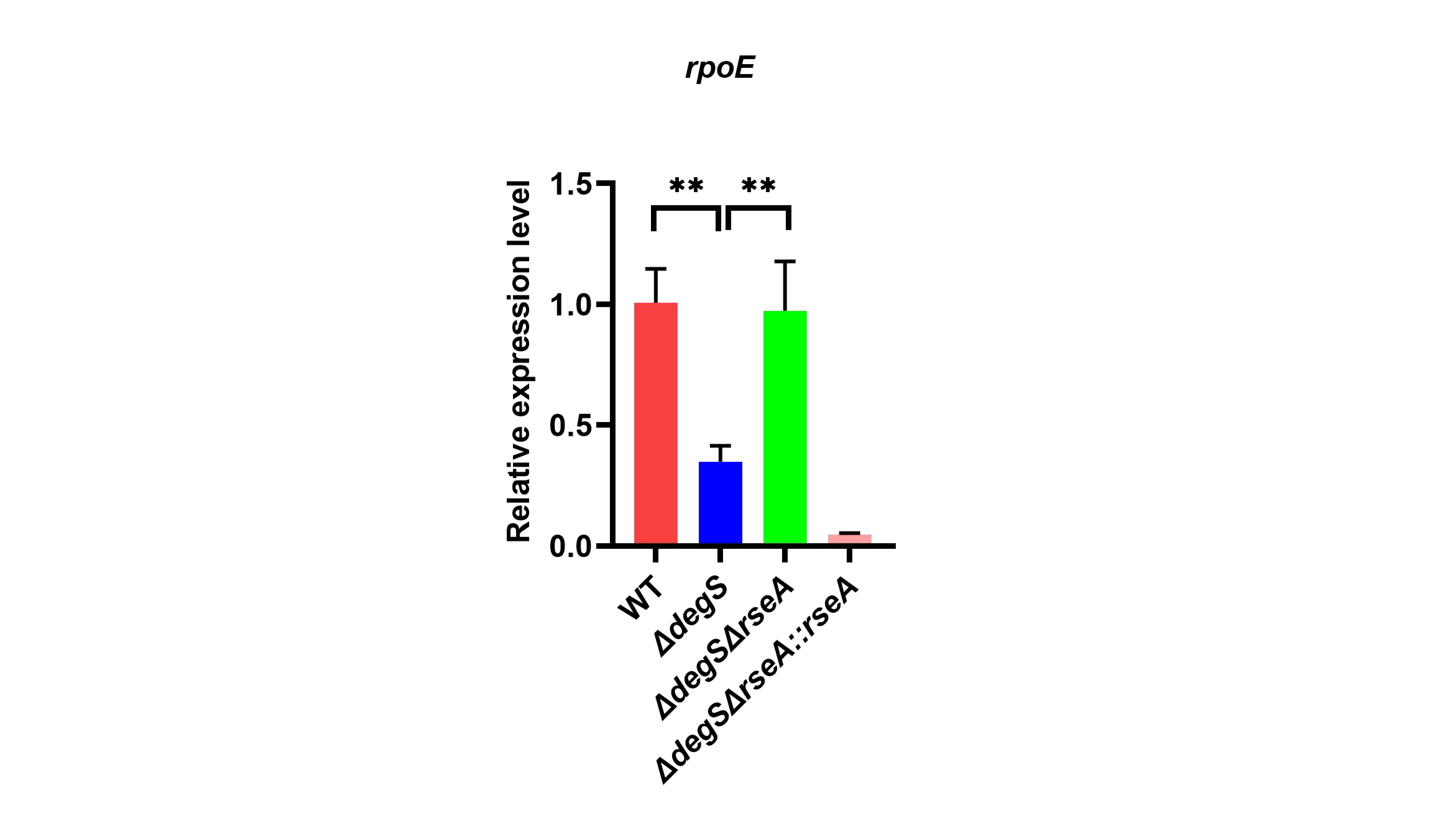

Supplement: Supplementary file 2 [file Image_2.TIF]

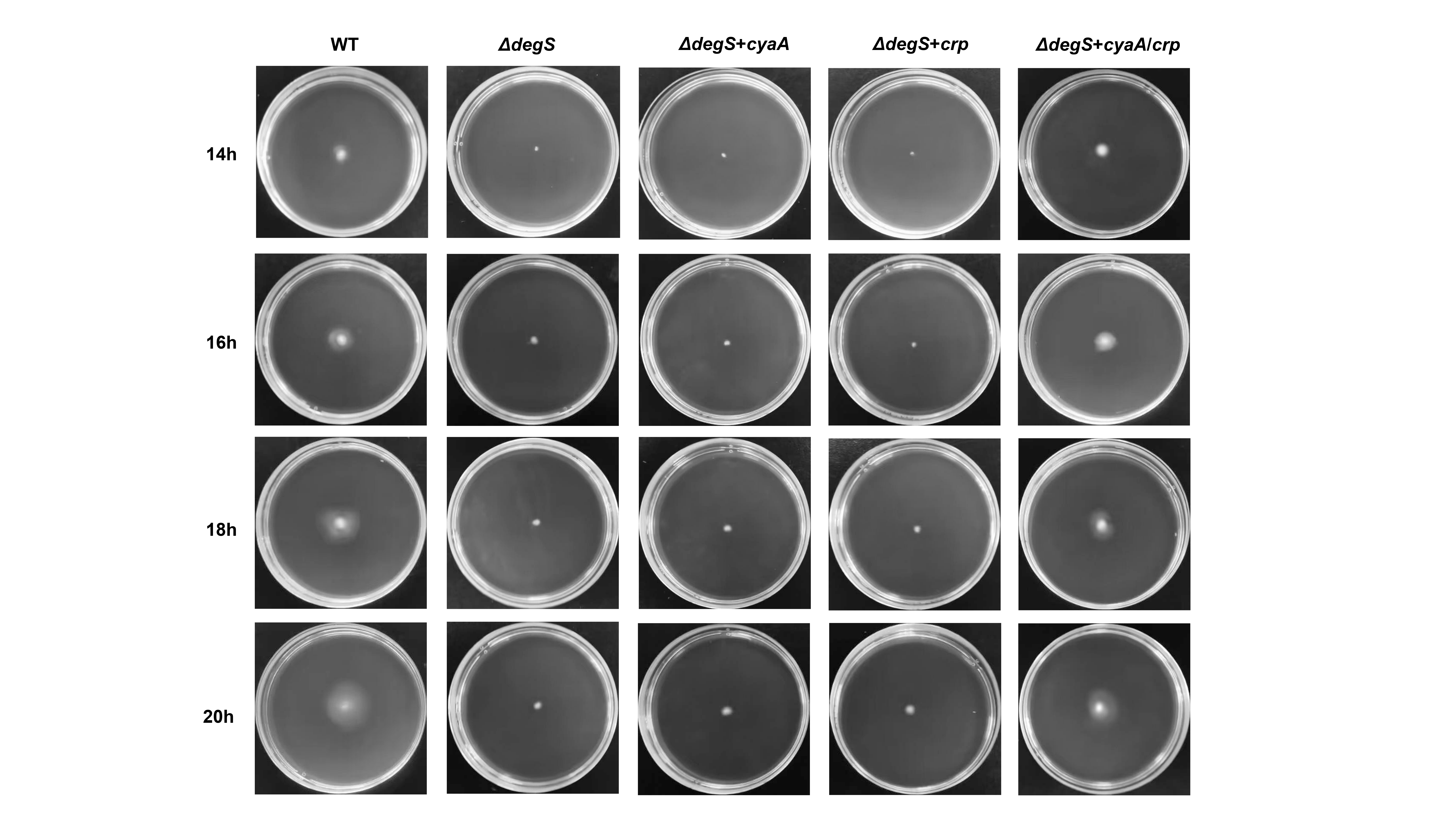

Supplement: Supplementary file 3 [file Image_3.TIF]

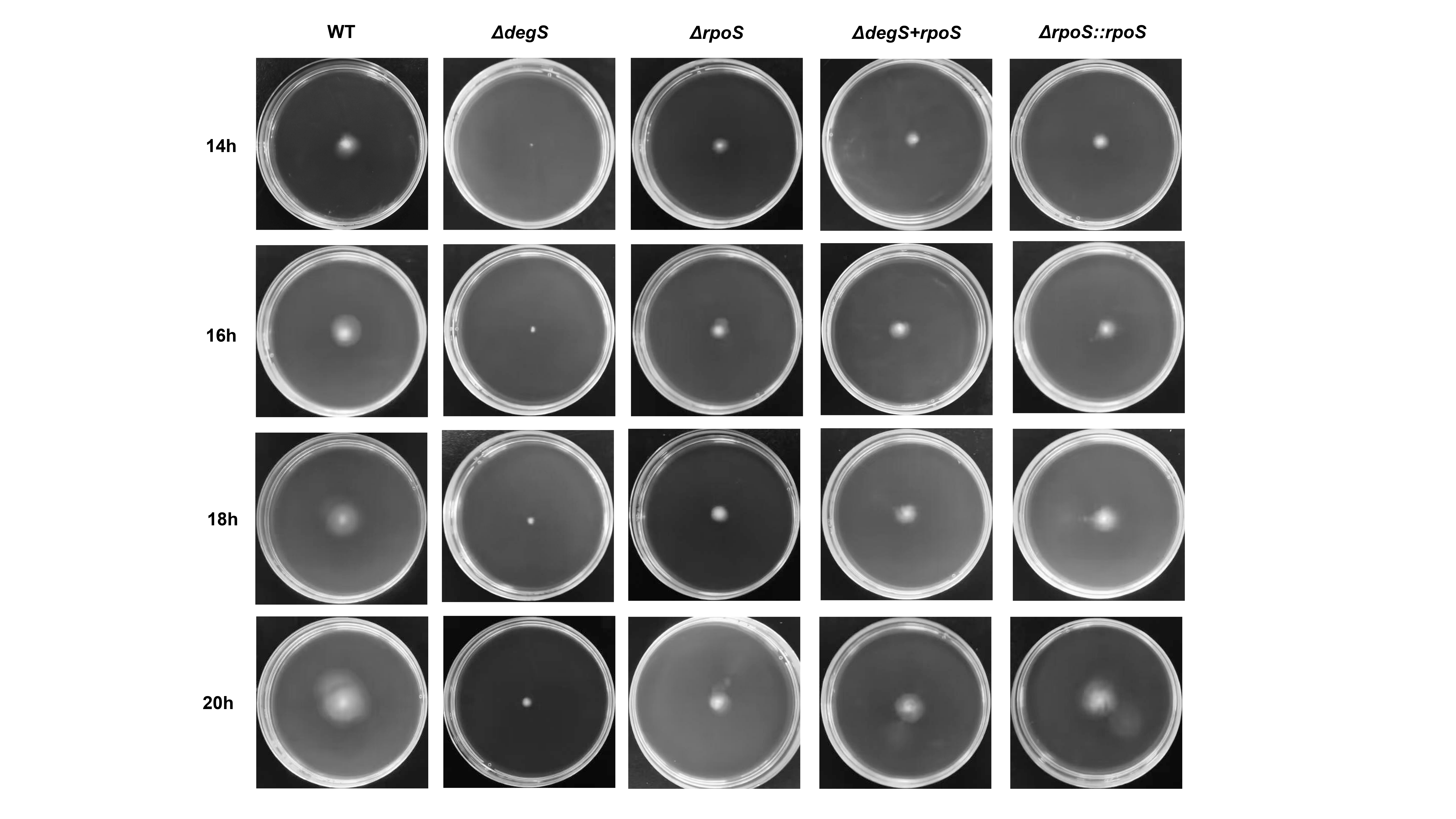

Supplement: Supplementary file 4 [file Image_4.TIF]

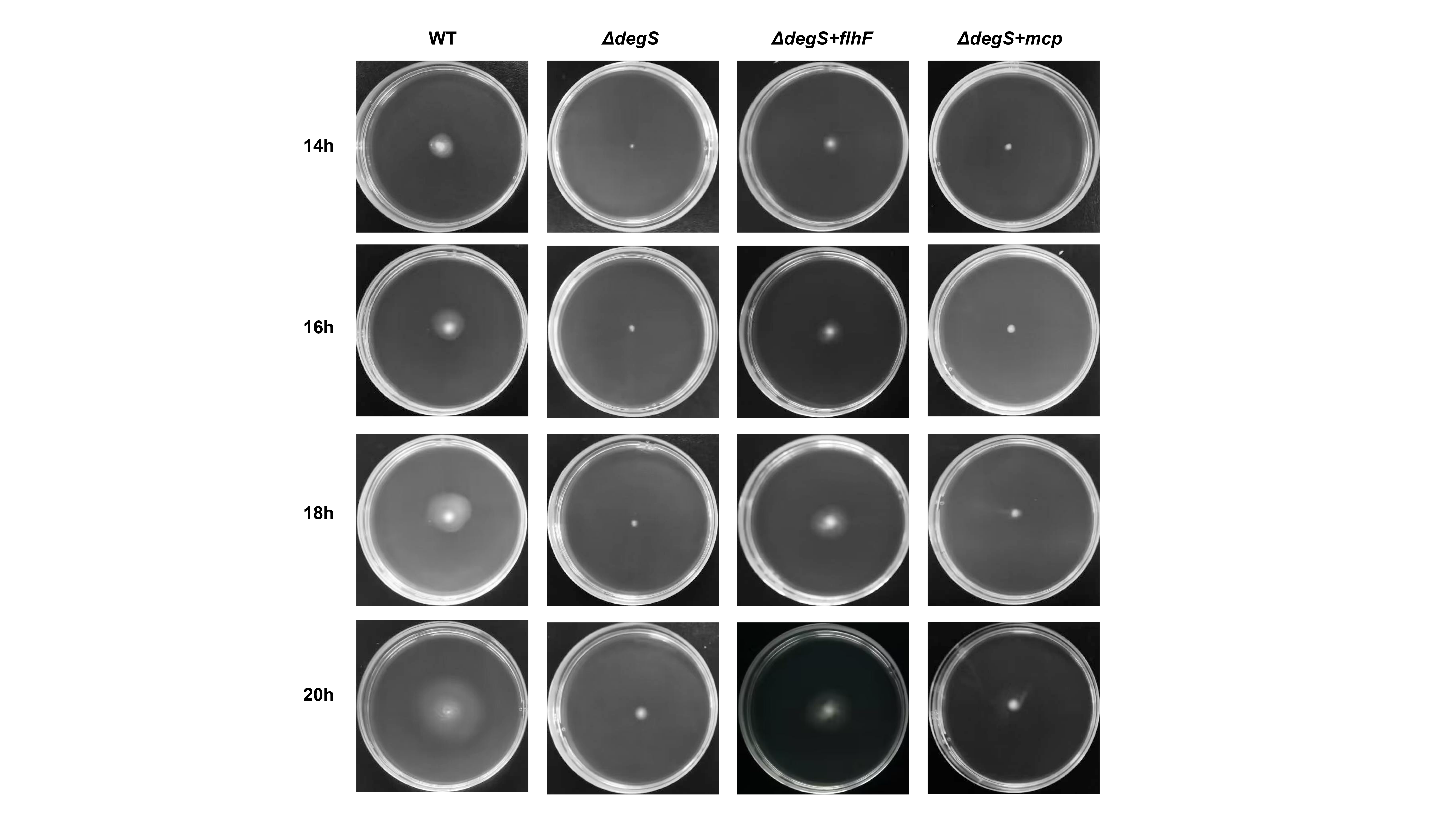

Supplement: Supplementary file 5 [file Image_5.TIF]
