# Supplementary material for: DegS protease regulates the motility, chemotaxis, and colonization of Vibrio cholerae
Source: Front Microbiol. 2023 Apr 5;14:1159986. doi: 10.3389/fmicb.2023.1159986 (PMC10113495; doi:10.3389/fmicb.2023.1159986)
Supplement: Supplementary file 6 [file Table_1.DOCX]

Supplementary Material

DegS Protease Regulates the Motility, Chemotaxis, and Colonization of *Vibrio cholerae*

Mei Zou^1,2†^, Kaiying Wang^1,2†^, Jiajun Zhao^1,2^, Huifang Lu^1,2^，Hui Yang^1,2^，Meirong Huang^2,3^，Lu Wang^1,2^， Guangli Wang^1,2^，Jian Huang^1,2*^ and Xun Min^1,2*^

*** Correspondence:** Jian Huang 81537648@qq.com； Xun Min minxunzmu@163.com.

# Supplementary Tables

**Supplementary Table 1. Bacterial strains and plasmids used in this study**

| Strain or plasmid | Genotype or feature(s) | Reference or source |
| --- | --- | --- |
| Strains |  |  |
| Non-O1/non-O139 *Vibrio cholerae* Strains |  |  |
| HN375 | Wild-type non-O1/non-O139 *V. cholerae* | Lab collections |
| *ΔdegS* | in-frame deletion of *degS* | Lab collections |
| *ΔdegS*+pBAD24 | *ΔdegS* complemented with pBAD24, Amp^r^ | This study |
| *ΔrpoE* | in-frame deletion of *rpoE* | This study |
| *ΔrpoE::rpoE* | *ΔrpoE* complemented with pBAD24-*rpoE*, Amp^r^ | This study |
| *ΔdegSΔrseA* | in-frame deletion of *degS* and *rseA* | This study |
| *ΔdegSΔrseA::rseA* | *ΔdegSΔrseA* complemented with pBAD24-*rseA*, Amp^r^ | This study |
| *ΔrpoS* | in-frame deletion of *rpoS* | This study |
| *ΔrpoS::rpoS* | *ΔrpoS* complemented with pBAD24-*rpoS*, Amp^r^ | This study |
| *Δcrp* | in-frame deletion of *crp* | This study |
| *ΔcyaA* | in-frame deletion of *cyaA* | This study |
| *ΔcyaAΔcrp* | in-frame deletion of *cyaA* and *crp* | This study |
| *Δcrp::crp* | *Δcrp* complemented with pBAD24-*crp*, Amp^r^ | This study |
| *ΔcyaA::cyaA* | *ΔcyaA* complemented with pBAD24-*cyaA*, Amp^r^ | This study |
| *Δcrp*+*pBAD24* | *Δcrp* complemented with pBAD24, Amp^r^ | This study |
| *ΔcyaA*+*pBAD24* | *ΔcyaA* complemented with pBAD24, Amp^r^ | This study |
| *ΔdegS*+*crp* | *ΔdegS* complemented with pBAD24-*crp*, Amp^r^ | This study |
| *ΔdegS*+*cyaA* | *ΔdegS* complemented with pBAD24-*cyaA*, Amp^r^ | This study |
| *ΔdegS*+*cyaA*/*crp* | *ΔdegS+cyaA* complemented with pBAD33-*crp*, Amp^r^and Chl^r^ | This study |
| *ΔdegS*+*flhF* | *ΔdegS* complemented with pBAD24-*flhF*, Amp^r^ | This study |
| *ΔdegS*+*mcp* | *ΔdegS* complemented with pBAD24-*mcp*, Amp^r^ | This study |
| *ΔdegS*+*rpoS* | *ΔdegS* complemented with pBAD24-*rpoS*, Amp^r^ | This study |
| *ΔcyaAΔcrp::cyaA* | *ΔcyaAΔcrp* complemented with pBAD24-*cyaA*, Amp^r^ | This study |
| *ΔcyaAΔcrp::crp* | *ΔcyaAΔcrp* complemented with pBAD24-*crp*, Amp^r^ | This study |
| *ΔcyaAΔcrp*+*pBAD24* | *ΔcyaAΔcrp* complemented with pBAD24, Amp^r^ | This study |
| Strains |  |  |
| DH5α | F−, ø80dlacZΔM15, Δ(lacZYA-argF) U169 deoR, recA1, endA1, hsdR17 (rk-,mk+), phoA, supE44, ʎ-, thi-1, gyrA96, relA1 | Lab collections |
| DH5α( λpir) | supE44 ΔlacU169 (ΦlacZΔM15) recA1 endA1 hsdR17 thi-1 gyrA96 relA1 λpir | Lab collections |
| WM3064 | thrB1004 pro thi rpsL hsdS lacZΔM15 RP4-1360 Δ(araBAD)567 ΔdapA1341::[erm pir] | Lab collections |
| Plasmids |  |  |
| pWM91 | Suicide plasmid; oriR oriT lacZ tetAR sacB, Amp^r^ | Lab collections |
| pWM91-*ΔrpoE* | pWM91 carrying upstream and downstream fragments flanking *rpoE*, Amp^r^ | This study |
| pWM91-*ΔcyaA* | pWM91 carrying upstream and downstream fragments flanking *cyaA*, Amp^r^ | This study |
| pWM91-*Δcrp* | pWM91 carrying upstream and downstream fragments flanking *crp*, Amp^r^ | This study |
| pWM91-*ΔrseA* | pWM91 carrying upstream and downstream fragments flanking *rseA*, Amp^r^ | This study |
| pWM91-*ΔrpoS* | pWM91 carrying upstream and downstream fragments flanking *rpoS*, Amp^r^ | This study |
| pBAD24 | Expression vector with araBAD promoter and rrnB T1 terminator, Amp^r^ | Lab collections |
| pBAD24-*rpoE* | pBAD24 expressing RpoE, Amp^r^ | This study |
| pBAD24-*rseA* | pBAD24 expressing RseA, Amp^r^ | This study |
| pBAD24-*crp* | pBAD24 expressing CRP, Amp^r^ | This study |
| pBAD24-*cyaA* | pBAD24 expressing cAMP, Amp^r^ | This study |
| pBAD24-*rpoS* | pBAD24 expressing RpoS, Amp^r^ | This study |
| pBAD24-*flhF* | pBAD24 expressing FlhF, Amp^r^ | This study |
| pBAD24-*mcp* | pBAD24 expressing MCP, Amp^r^ | This study |
| pBAD33 | Expression vector with araC promoter and rrnB T1 terminator, Chl^r^ | Lab collections |
| pBAD33-*crp* | pBAD33 expressing CRP, Chl^r^ | This study |

**Supplementary Table 2 Primers used in this study**

| Name | Primer sequence (forward/reverse, 5’ to 3’) | Use and description |
| --- | --- | --- |
| Primers for mutant construction | | |
| *rpoE* up-F | CCGCTCGAGCGGCAATAAAGCGCACGGTT | For construction of deletion mutant of *∆rpoE* |
| *rpoE* up-R | ACCATAGTCATTACGGAATTTGCGTCGAGCGGTCACTCCTATTGTTAT |  |
| *rpoE* down-F | ATAACAATAGGAGTGACCGCTCGACGCAAATTCCGTAATGACTATGGT |  |
| *rpoE* down-R | TCCCCCGGGTACAACAACGCCTCTGCAGACTCT |  |
| *rpoE* -F | TCCCCCGGGATGAACGAGCAACTGACCGATC | For cloning complete length of *rpoE* into pBAD24 |
| *rpoE* -R | CCCAAGCTTTTACAGAAGAGGTTTGATTTTCTTC |  |
| *rseA* up-F | CCCTCGAGCGAATGAACGAGCAACTGACC | For construction of deletion mutant of *∆degS∆rseA* |
| *rseA* up-R | CTGGGTGTTGGTGAGAAGCACTATTCTGATCCTGAGCCAATCCTAAA |  |
| *rseA* down-F | TTTAGGATTGGCTCAGGATCAGAATAGTGCTTCTCACCAA CACCCAG |  |
| *rseA* down-R | CGGGATCCGGCTCAACTCCGCCACATCC |  |
| *rseA* -F | CGGAATTCCGATGACTATGGTGAATAGAATGGCTGAC | For cloning complete length of *rseA* into pBAD24 |
| *rseA* -R | GCTCTAGAGCTCATTCCACAACCGATTCAACTG |  |
| *rpoS* up-F | CGCG^GATCCGGATGAGAAGATAGCGAAG | For construction of deletion mutant of *∆rpoS* |
| *rpoS* up-R | TCATAGCCAAGAAGCCCAACAGTAAGGAGCGGTGAAAA |  |
| *rpoS* down-F | TTTTCACCGCTCCTTACTGTTGGGCTTCTTGGCTATGA |  |
| *rpoS* down-R | CCGC^TCGAGACCGAACTGCCGAATGTGT |  |
| *crp* up-F | CGCGGATCCTCAATGGACGACTACCTC | For construction of deletion mutant of  *∆crp* and *ΔcyaAΔcrp* |
| *crp* up-R | TCTTGGCGAGTGATCTTGCAACTGAACCTTTTACGA |  |
| *crp* down-F | TCGTAAAAGGTTCAGTTGCAAGATCACTCGCCAAGA |  |
| *crp* down-R | CCGCTCGAGGCACAAGTTCAGCCACAA |  |
| *cyaA* up-F | CGCGGATCCAGTATCCAGAATCACATCGC | For construction of deletion mutant of  *∆cyaA* and *ΔcyaAΔcrp* |
| *cyaA* up-R | TTGCGAAGGCTTGGAATACTATCAGCAGAAAAATAACCG |  |
| *cyaA* down-F | CGGTTATTTTTCTGCTGATAGTATTCCAAGCCTTCGCAA |  |
| *cyaA* down-R | ATAAGAATGCGGCCGCCAGCCCACAACTGACCATC |  |
| Primers for constructs for complementation | | |
| *crp* -F | CCGGAATTCATGGTTCTAGGTAAACCTCAAACC | For cloning complete length of *crp* into pBAD24 |
| *crp* -R | CCCAAGCTTTTAGCGAGTGCCGTAAACCAC |  |
| *crp* -F | GCTCTAGAATGGTTCTAGGTAAACCTCAAACC | For cloning complete length of *crp* into pBAD33 |
| *crp* -R | CCCAAGCTTTTAGCGAGTGCCGTAAACCAC |  |
| *cyaA*-F | CCGGAATTCTTGCAGGCTTATACTCAGACC | For cloning complete length of *cyaA* into pBAD24 |
| *cyaA* -R | CCCAAGCTTTTAGGCATTGACCACTTG |  |
| *rpoS*-F | CGGAATTCATGAGTGTCAGCAATACCGTAACCAAAGT | For cloning complete length of *rpoS* into pBAD24 |
| *rpoS*-R | GCGAAGCTTTTAGTTGTCGTATTCGACGTTAAACAGC |  |
| *flhF*-F | GCTCTAGATTGAAAATAAAACGATTTTTTGCCAAG | For cloning complete length of *flhF* into pBAD24 |
| *flhF* -R | CCCAAGCTTCTAGAATCTCTCTGAATCACTGGTCCA |  |
| *mcp* -F | GCTCTAGAATGATGGTATCCAATCAACATAACAATG | For cloning complete length of *mcp* into pBAD24 |
| *mcp*-R | CCCAAGCTTTCAGGCATTGTAACGCGCGACT |  |
| Primers for Real time PCR | | |
| q*-cyaA-*F | GCCGCTGTTTCTATCTCAA | For qRT-PCR to targeting *cyaA* |
| q*-cyaA-*R | TCAGTTCCCGTAACGCTTC |  |
| q-*crp-*F | ACCCATCAAAAAGCACACTG | For qRT-PCR to targeting *crp* |
| q*-crp-*R | GCTCACCGATAAAATCACCT |  |
| q*-rpoS-*F | ATCCGTGCGGTTGAGAAA | For qRT-PCR to targeting *cyaA* |
| q*-rpoS-*R | TCGGTAGACGAATGGTGC |  |
| q*-mshB-*F | GCCACTGGTGTTCTCTCT | For qRT-PCR to targeting *mshB* |
| q*-mshB-*R | AGTCGCATCATCGGTATT |  |
| q*-mcp-*F | CGCAATCCATCAGTTCACC | For qRT-PCR to targeting *mcp* |
| q*-mcp-*R | CGGCTTGTTCTGCTTCTTC |  |
| q*-cheB1-*F | ACGGTATTTCCGCTGTCC | For qRT-PCR to targeting *cheB1* |
| q*-cheB1-*R | GCATCCAATGTGGCTTTT |  |
| q*-flhF-*F | TCTATGACTCAACGCTTTGCTAATA | For qRT-PCR to targeting *flhF* |
| q*-flhF-*R | GACGCTCTGAACTACTCTGACTACG |  |
| q*-flaG-*F | TTTCGCTTTCAGGGGATAA | For qRT-PCR to targeting *flaG* |
| *q-flaG-*R | GTCTCTGCCGATTCTTGCT |  |
| q*-flaD-*F | CGGCAGAAGGCAAAGACAA | For qRT-PCR to targeting *flaD* |
| q*-flaD-*R | AGCGTTCACGGTAATGGTT |  |
| q*-flaC-*F | AGGTTTAGGTGTCGCCGTTC | For qRT-PCR to targeting *flaC* |
| q*-flaC-*R | GGTGGTCTCTTTCATTGCCC |  |
| q*-cheR1-*F | GTCTGACTTGTTGCGGGATGT | For qRT-PCR to targeting *cheR1* |
| q*-cheR1-*R | AAACCGTAAATGGGTAGGAAT |  |
| q*-flgA-*F | ATGAAGAAACCAGAGAAACGAAA | For qRT-PCR to targeting *flgA* |
| q*-flgA-*R | AAATAATAAAAAGCCGATAAAGC |  |
| q*-rpoE-*F | TATCGAACCCGGAGAACATT | For qRT-PCR to targeting *rpoE* |
| q*-rpoE-*R | TTTCCTCGTAACTCAAGCCA |  |
| q-16sRNA-F | CGGTAATACGGAGGGTGCAA | For qRT-PCR to targeting 16sRNA |
| q-16sRNA-R | CACCTGCATGCGCTTTACG |  |

**Supplementary Table 3. Differentially expressed motility-related genes between WT and *ΔdegS***

| Gene | Description | Log2FC | FDR | Style |
| --- | --- | --- | --- | --- |
| *cyaA* | adenylate cyclase | -0.810263158 | 6.83933E-11 | down |
| VC2614(*crp*) | cAMP-regulatory protein | -1.560057984 | 0 | down |
| VC0534(*rpoS*) | RNA polymerase sigma-38 factor | -2.099560766 | 1.01229E-11 | down |
| VC1643(*mcp*) | methyl-accepting chemotaxis protein | -0.591826811 | 4.00912E-06 | down |
| VC2068(*flhF*) | flagellar biosynthesis regulator FlhF | -0.675168319 | 0.004175067 | down |
| VC2203(*flgA*） | flagellar basal body P-ring biosynthesis protein FlgA | -1.051005671 | 2.86438E-14 | down |
| VC2187(*flaC*） | flagellin | -0.82666004 | 3.89359E-07 | down |
| VC2143(*flaD*） | flagellin | -1.015087023 | 1.93212E-12 | down |
| VC2141(*flaG*） | flagellar protein FlaG | -1.03652733 | 5.15749E-05 | down |
| VC0408(*mshB*) | MSHA pilin protein MshB | -0.639969947 | 0.045598548 | down |
| VC2062(*cheB1*) | chemotaxis-specific methylesterase | -0.697389549 | 0.012306298 | down |
| VC2201(*cheR1*) | chemotaxis protein CheR | -0.668006472 | 0.00010905 | down |
